# Long-term symptom burden in a young, ambulatory cohort after the omicron outbreak in China

**DOI:** 10.3389/fpubh.2025.1702599

**Published:** 2025-12-18

**Authors:** Simeng Ren, Yumeng Tan, Hongkun Xu, Mian Wang, Rumei Xiang, Jiayue Jin, Baojin Han, Jiaheng Shi, Jingyu Zhang, Jinliang Yang, Xin Tian, Ying Li, Jiaojiao Chen, Wenzheng Zhang, Jingqi Yang, Xin Shelley Wang, Qiuling Shi, Jie Liu

**Affiliations:** 1 Guang’anmen Hospital, China Academy of Chinese Medical Sciences, Beijing, China; 2 China Center for Evidence-Based Traditional Chinese Medicine, China Academy of Chinese Medical Sciences, Beijing, China; 3 Graduate School, Beijing University of Chinese Medicine, Beijing, China; 4 Department of Traditional Chinese Medicine, Xijing Hospital, Air Force Medical University, Xi'an, China; 5 School of Public Health, Chongqing Medical University, Chongqing, China; 6 Department of Social Medicine and Health Management, School of Public Health, Lanzhou University, Lanzhou, China; 7 Department of Symptom Research, The University of Texas MD Anderson Cancer Center, Houston, TX, United States

**Keywords:** COVID-19, long-term symptom burden, patient-reported outcome, symptom clusters, influencing factors

## Abstract

**Introduction:**

At the end of 2022, an outbreak of the Omicron BF.7 and BA.5.2 subvariants of SARS-CoV-2 occurred in China. In this prospective cohort study, we investigated the pattern of development of major symptom burden and influencing factors in infected Chinese patients.

**Methods:**

First-time infected outpatients were enrolled from December 7, 2022, to January 11, 2023 (*N* = 355). The prevalence of symptoms was monitored by a repeated patient-reported quantitative symptom survey over nine months.

**Results:**

At the onset of the infection, the most prevalent symptoms (score ≥1 on a 0–10 numeric rating scale) were fatigue (91.8%), cough (91.8%), and sore throat (91.5%) among 33 symptoms monitored. Patients with higher scores for symptom Cluster II (lack of appetite, disturbed sleep, shivering, drowsiness, sweating, nausea, depression, and anxiety) and symptom cluster V (fatigue, sore throat, dry mouth, and dizziness) reported poorer quality of life than other patients during the first month after enrolment. The most severe symptoms (score≥7) lasted during 3–9 months were depression (5.2%), fatigue (4.8%), anxiety (4.8%), runny nose (4.3%), muscle or joint pain (3.3%), nasal congestion (3.0%), disturbed sleep(2.6%). Younger age, female sex, and body mass index of at least 24 kg/m^2^ predicted more severe baseline symptoms and slower resolution (all *p* < 0.01).

**Conclusion:**

This cohort study identified patterns and characteristics of symptom evolution in outpatients at 9 months post-COVID-19 diagnosis and provides targets for long-term care.

## 1 Introduction

Since the outbreak of the COVID-19 pandemic in December 2019, SARS-CoV-2 has infected hundreds of millions worldwide, leading to more than 15.9 million deaths. In particular, the mortality rate among individuals aged 15 and above has increased by 22% in men and 17% in women, and the global average life expectancy has been reduced by 1.6 years (1). As a persistent and relapsing respiratory illness, COVID-19 has entered a new normal characterized by wave-like epidemics, posing long-term challenges to global public health systems and socio-economic stability.

Patients with COVID-19 may present prolonged multisystem involvement and significant disability. Long COVID is an infection-associated chronic condition that occurs after SARS-CoV-2 infection and is present for at least 3 months as a continuous, relapsing and remitting, or progressive disease state that affects one or more organ systems (2). Previous studies (3) have shown that one-third of non-hospitalized patients still experience symptoms and are unable to return to work 22 months after the onset of COVID-19. A study conducted in China (4) indicated that 10–30% of individuals have experienced one or more Long COVID symptoms 12 months subsequent to their initial infection. The most common symptoms include fatigue (30.53%), memory impairment (27.93%), reduced physical ability (18.29%), and brain fog (16.87%). It can impair patients’ ability to work, attend school, take care of family, and care for themselves, exerting profound physical and emotional impacts on the patients, their families, and caregivers. Symptom clusters were defined as two or more related symptoms that may vary over time (5). Compared to a single symptom, symptoms within a cluster may share common mechanisms or underlying causes, and the presence of a symptom cluster may lead to different outcomes than individual symptoms alone.

In December 2022, China experienced a severe outbreak of Omicron infection. The dominant subvariants were BA.5.2.1.7 (BF.7), BA.5.2 and their sublineages. With the advancement of large-scale vaccination, the pathogenicity of SARS-CoV-2 has gradually weakened, and hospitalization of infected individuals is now uncommon (6). Young adults, as a core component of the workforce, account for 80–97% of all non-hospitalized COVID-19 cases (7, 8). Previous studies have shown differences in the prevalence of Long COVID symptoms and their distribution between hospitalized and non-hospitalized patients (9, 10). Current Long-COVID research primarily examines the incidence of individual symptoms, with limited emphasis on longitudinal symptom cluster patterns after acute COVID-19, and lacks quantitative patient-reported outcome (PRO) measures.

This study investigated symptom burdens in these outpatients over a one-year period, and described the characteristics, evolution, and differentiated patterns of symptom clusters in patients with first-time SARS-CoV-2 infection involving multiple organ systems, as well as explored whether there exists a core symptom cluster that most impacts quality of life. The aim of this study is to deepen the understanding of Long COVID associated with Omicron, provide a basis for clinical treatment decisions, and valuable insights for the development of treatment and prevention strategies for this patient population.

## 2 Methods

### 2.1 Study design and participants

In this prospective cohort study, 355 outpatients with first-time confirmed SARS-CoV-2 positivity were recruited from seven regions in China via the social media platform WeChat from December 7, 2022, to January 11, 2023. The inclusion criteria for the patients in this study were (1) SARS-CoV-2 infection confirmed either within 7 days of the first positive antigen or nucleic acid test, or according to the presence of typical COVID-19 symptoms (e.g., fever and/or respiratory symptoms) and a history of living with a patient with confirmed COVID-19 within 7 days; (2) education level of third grade or higher and ability to fully participate in the study, and (3) understanding of the study aims and voluntary participation. The exclusion criteria were (1) hospitalization at enrollment; (2) severe, life-threatening organic disease (including COVID-19 and other conditions); (3) patients deemed unsuitable by the researchers, and (4) cognitive dysfunction.

Patients were requested to complete a questionnaire to report the date of the first positive nucleic acid or antigen test, the date of onset of possible COVID-19-related symptoms, demographic characteristics and clinical variables, and types and severity of symptoms.

Information regarding patients’ self-reported symptoms was collected using an electronic data capture system. Given the lack of outcome assessment tools for Chinese patients with COVID-19, a comprehensive list of 33 COVID-19–related symptoms were developed by this Chinese study team based on guidelines for developing PRO tools (11, 12). The tool exhibited good reliability (Cronbach’s *α* coefficient = 0.92) and criterion validity, as validated using the EuroQol five-dimension, five-level (EQ-5D-5L) (used as an anchor; *r* = 0.30; *p* < 0.01).

The severity of the patients’ symptoms was assessed using an 11-point Likert scale (0–10), where a score of 0 indicated “not present” and 10 indicated “as bad as you can imagine,” with a 24-h recall period. After study enrollment, patients reported their symptoms via an electronic outcome portal daily for 10 consecutive days post-enrollment, at weeks 2 and 3, and at the start of months 1–9.

### 2.2 Ethics

All participants were required to complete an electronic informed consent form before filling out the questionnaire. This study was approved by the Ethics Committee of the Institute of Basic Research in Clinical Medicine, China Academy of Chinese Medical Sciences (P22001/PJ01). All personal data were de-identified prior to data analysis. The reporting of the study findings followed the recommendations of the Strengthening the Reporting of Observational Studies in Epidemiology (STROBE) guidelines (13).

### 2.3 Statistical methods

Statistical analyses were performed using the SPSS Premium Student GradPack for Windows (version 29.0; IBM), SAS software (version 9.4; SAS Institute Inc.), and the R software environment (version 4.0.2). To describe patients’ demographic and clinical characteristics, continuous data were summarized as mean ± standard deviation (SD) for normally distributed data, or median and interquartile range (IQR) for non-normally distributed data. Categorical data were summarized as frequencies and percentages. To evaluate symptom changes during the acute phase (within 1 month after enrollment), nonlinear mixed-effect models were employed to profile the evolution of each symptom over time. Longitudinal symptom clustering was performed using two model parameters: the intercept, which represented the estimated initial level of symptoms, and the change point, which indicated the time at which the symptom’s slope transitioned from one segment to another. For each cluster obtained, a composite score was calculated by averaging the scores of symptoms within the cluster. Symptom clusters significantly impacting quality of life as measured using the EuroQol visual analogue scale (EQ-VAS) were identified using a linear mixed-effects model that included all composite cluster scores and the time points (day 1 and weeks 1, 2, 3, and 4 after recruitment) with adjustment for age, sex, body mass index (BMI), comorbidities, and COVID-19 vaccine doses received (≥3 doses vs. <3 doses). Furthermore, a multivariable mixed-effects model was used to evaluate the effect of patients’ baseline demographic and clinical characteristics on cluster scores during the first 30 days and symptoms scores from 3 months to 9 months of the study. Using two-sided statistical testing at the 0.05 significance level.

## 3 Results

### 3.1 Demographic and clinical characteristics of the study population

**Figure 1:**
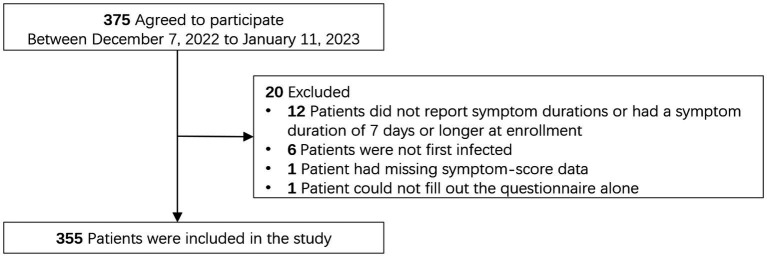
Patient exclusion flowchart.

We enrolled 355 patients in the study from December 7, 2022, to January 11, 2023 (Figure 1). As shown in Table 1, the mean age of participants was 31.9 years and 96.3% were younger than 60 years of age; 69% were female. The COVID-19 vaccination rate was 95.5%, with 75.5% of the participants receiving at least three doses. Only four (1.6%) women were pregnant. Most of the participants (87%) reported no comorbidities, 12 (3.4%) had a history of respiratory diseases, 9 (2.5%) patients had cardiovascular or cerebrovascular disease, 5 (1.4%) had diabetes, 5 (1.4%) had chronic liver or kidney diseases, 3 (0.9%) had cancer, 1 (0.3%) had immunodeficiency, and 17 (4.8%) had other diseases. At enrollment, the mean (± SD) duration of symptoms was 2.53 ± 1.35 days, and the mean (± SD) number of days since COVID-19 diagnosis was 1.43 ± 1.41 days.

**Table 1:** Patient demographic and clinical characteristics.

Characteristics	No.	Percentage (%)
Age, years		
<18	8	2.3
18–39	271	76.3
40–59	63	17.7
≥60	13	3.7
Sex		
Female	245	69.0
Male	110	31.0
Vaccinated against SARS-CoV-2, dose		
0	16	4.5
1	12	3.4
2	59	16.6
≥3	268	75.5
Comorbidities		
None	309	87.3
Other diseases	17	4.8
Respiratory diseases	12	3.4
Cardiovascular or cerebrovascular diseases	9	2.5
Diabetes	5	1.4
Chronic liver or kidney disease	5	1.4
Cancer	3	0.9
Immunodeficiency	1	0.3
Not reported	1	0.3
Heavy smoker		
No (not a smoker or smokes ≤60 packs/year)	340	95.8
Yes (smokes >60 packs/year)	15	4.2
Body mass index, kg/m^2^		
<18.5	33	9.3
18.5–23.9	213	60.0
24.0–27.9	85	23.9
≥28.0	24	6.8
Region^a^		
North China	115	32.4
East China	103	29.0
Southwest China	61	17.2
Northwest China	31	8.7
South China	30	8.5
Central China	11	3.1
Northeast China	4	1.1

a Northeast China: Heilongjiang Province, Jilin Province, Liaoning Province. North China: Beijing, Tianjin, Shanxi Province, Hebei Province, and the Inner Mongolia Autonomous Region. East China: Shanghai Municipality, Jiangsu Province, Zhejiang Province, Anhui Province, Jiangxi Province, Shandong Province, Fujian Province, and Taiwan Region of China. South China: Guangdong Province, the Guangxi Zhuang Autonomous Region, Hainan Province, the Hong Kong Special Administrative Region, and the Macao Special Administrative Region. Central China: Henan Province, Hubei Province, and Hunan Province. Northwest China: Shaanxi Province, Gansu Province, Qinghai Province, the Ningxia Hui Autonomous Region, and the Xinjiang Uygur Autonomous Region. Southwest China: Chongqing Municipality, Sichuan Province, Guizhou Province, Yunnan Province, and the Tibet Autonomous Region.

### 3.2 Symptom prevalence and influencing factors of long-term symptoms

At enrollment, among the 33 screened symptoms, the 10 most prevalent symptoms (Likert scale score ≥ 1) were fatigue (91.8%), coughing (91.8%), sore throat (91.5%), dry mouth (90.4%), sputum (85.9%), feeling cold (85.9%), throat itching (85.3%), muscle or joint pain (84.2%), nasal congestion (82.4%), and headache (81.6%). In comparison, the most frequent moderate to severe (Likert scale score ≥ 4) symptoms were fatigue (71.8%), fever (68.1%), and dry mouth (66.3%). The most frequent severe (Likert scale score ≥ 7) symptoms were fever (46.0%), fatigue (39.5%), and muscle or joint pain (37.0%) (Table 2).

**Table 2:** Symptom prevalence at enrollment, month 1 to month 9 (%).

Symptom	Enrollment			Month 1			Month 2		
	Score ≥1	Score ≥4	Score ≥7	Score ≥1	Score ≥4	Score ≥7	Score ≥1	Score ≥4	Score ≥7
Fatigue	91.8	71.8	39.5	37.2	7.2	2.1	38.2	8.5	1.6
Coughing	91.8	60.6	25.5	31.8	4.8	1.0	14.1	3.6	0.0
Sore throat	91.5	59.1	31.5	7.3	0.7	0.0	4.9	0.8	0.0
Dry mouth	90.4	66.3	31.4	23.1	2.4	0.0	20.6	3.2	0.0
Sputum	85.9	50.6	14.1	29.7	3.4	0.3	14.1	2.0	0.0
Feeling cold	85.9	61.6	30.8	12.4	2.4	0.3	9.3	1.2	0.0
Throat itching	85.3	49.3	21.8	16.6	2.4	0.3	10.5	3.6	0.8
Muscle or joint pain	84.2	64.1	37.0	12.8	2.8	1.4	17.3	2.8	0.0
Nasal congestion	82.4	57.2	23.8	13.4	1.0	0.0	8.1	1.6	0.0
Headache	81.6	63.3	36.4	10.0	1.7	0.7	11.0	1.2	0.0
Runny nose	81.0	44.8	17.8	11.7	1.4	0.0	8.1	1.6	0.0
Dizziness	80.4	56.3	27.3	12.8	2.4	0.0	11.4	0.8	0.4
Lack of appetite	79.0	55.0	24.6	13.1	1.4	0.3	8.9	1.6	0.4
Fever	78.8	68.1	46.0	2.1	1.0	0.3	1.2	0.4	0.4
Disturbed sleep	69.2	45.5	21.8	18.3	5.5	2.1	19.0	4.4	1.2
Shivering	65.4	43.9	19.8	5.6	2.1	0.3	2.8	0.4	0.0
Drowsiness	64.3	38.5	17.3	16.6	5.2	1.4	19.0	3.2	0.8
Sweating	62.4	38.5	14.5	7.6	1.7	0.3	6.0	1.6	0.4
Nausea	56.3	32.1	10.8	7.9	0.7	0.0	5.6	0.8	0.4
Depression	55.4	31.8	10.8	14.8	4.5	1.0	16.6	3.6	0.8
Change in taste	55.1	28.4	12.2	6.9	0.7	0.3	4.8	0.0	0.0
Shortness of breath	50.8	22.6	4.2	20.0	1.7	0.3	13.8	1.6	0.0
Anxiety	50.7	27.2	10.5	15.2	3.8	1.4	18.3	3.7	1.2
Chest tightness	49.0	25.2	3.7	17.6	1.4	0.0	11.7	1.2	0.0
Change in sense of smell	46.3	23.9	9.4	6.9	1.4	0.3	6.9	0.4	0.0
Racing heartbeat	45.7	16.0	3.1	15.2	3.8	0.3	17.3	3.6	0.8
Chest pain	40.2	13.0	2.0	9.3	1.4	0.7	8.1	0.8	0.0
Constipation	35.8	18.5	5.7	12.8	2.4	0.3	8.1	0.8	0.4
Vomiting	35.1	19.3	7.6	3.1	0.3	0.0	3.6	0.4	0.0
Diarrhea	33.3	12.1	2.0	6.2	0.7	0.3	6.9	2.0	0.0
Bloating	31.2	14.2	2.8	7.6	1.0	0.0	8.5	0.8	0.4
Pain in the abdomen	31.2	11.3	1.7	5.2	1.0	0.3	3.6	0.0	0.0
Rash	16.0	6.9	2.0	7.6	1.0	0.7	6.0	0.8	0.4

Symptom	Month 3			Month 6			Month 9		
	Score ≥1	Score ≥4	Score ≥7	Score ≥1	Score ≥4	Score ≥7	Score ≥1	Score ≥4	Score ≥7
Fatigue	37.4	10.9	2.9	37.9	9.9	1.2	39.7	9.9	0.7
Coughing	18.9	3.8	0.4	16.9	2.5	1.3	15.7	3.6	0.7
Sore throat	10.1	2.1	0.8	14.3	3.1	0.0	11.5	2.9	1.4
Dry mouth	24.4	4.2	0.0	29.2	5.0	1.2	22.1	2.1	0.7
Sputum	18.1	3.4	0.0	19.3	3.1	0.6	14.9	2.8	0.7
Feeling cold	13.9	2.1	0.4	13.0	1.9	0.0	13.5	2.1	0.0
Throat itching	14.3	2.1	0.4	15.5	1.9	0.0	15.7	2.9	0.7
Muscle or joint pain	13.9	4.2	1.3	18.6	5.6	0.6	15.6	5.0	1.4
Nasal congestion	14.0	2.1	0.4	15.5	2.5	1.9	14.3	2.1	0.7
Headache	10.9	2.1	0.0	15.0	3.8	1.3	12.8	2.8	0.7
Runny nose	15.5	2.9	0.4	16.8	3.1	2.5	16.3	2.1	1.4
Dizziness	8.8	2.1	0.0	18.6	5.0	1.9	17.0	2.8	0.0
Lack of appetite	9.7	1.3	0.4	15.5	3.1	0.0	12.8	1.4	0.7
Fever	2.5	1.3	0.8	4.3	1.9	1.2	2.8	0.0	0.0
Disturbed sleep	17.7	5.9	1.3	20.5	4.3	0.6	24.1	5.7	0.7
Shivering	6.3	0.4	0.0	3.7	1.2	0.6	2.1	0.0	0.0
Drowsiness	18.5	3.4	1.3	23.6	5.0	1.2	18.4	3.5	0.0
Sweating	10.1	2.9	0.8	13.1	5.0	1.3	15.0	1.4	0.0
Nausea	5.9	0.8	0.4	8.7	2.5	0.6	9.2	2.8	0.0
Depression	19.5	4.2	1.3	22.4	7.5	2.5	22.0	6.4	1.4
Change in taste	3.4	0.4	0.0	6.8	1.9	1.2	6.4	0.0	0.0
Shortness of breath	15.3	2.1	0.8	14.9	3.1	0.6	14.2	0.7	0.0
Anxiety	21.8	6.3	2.1	25.6	8.8	1.3	21.4	6.4	1.4
Chest tightness	15.5	1.3	0.0	12.4	1.2	0.6	14.9	0.7	0.0
Change in sense of smell	3.8	0.4	0.0	6.8	2.5	1.9	6.4	0.0	0.0
Racing heartbeat	15.5	2.9	0.0	14.3	2.5	0.0	12.1	1.4	0.7
Chest pain	7.1	0.4	0.0	10.6	1.2	0.0	6.4	0.0	0.0
Constipation	8.0	1.7	0.0	10.6	1.9	0.0	10.6	1.4	0.7
Vomiting	2.1	0.4	0.0	5.0	0.6	0.0	2.1	0.0	0.0
Diarrhea	6.7	0.8	0.0	11.2	1.9	0.0	13.6	3.6	0.0
Bloating	9.3	1.3	0.0	9.3	3.1	0.6	8.5	0.7	0.0
Pain in the abdomen	5.9	1.7	0.4	8.8	0.6	0.0	11.3	1.4	0.7
Rash	5.0	2.1	0.8	9.3	1.9	0.0	7.8	1.4	0.0

Compared with male patients, female patients were more likely to report higher severity levels for long-term fatigue, disturbed sleep and muscle or joint pain (*p* < 0.05). Patients with a BMI ≥ 24 reported higher severity levels for long-term disturbed sleep, muscle or joint pain, runny nose and nasal congestion compared to those with a BMI < 24 (*p* < 0.05).

### 3.3 Longitudinal evolution of symptoms and associated factors

**Figure 2:**
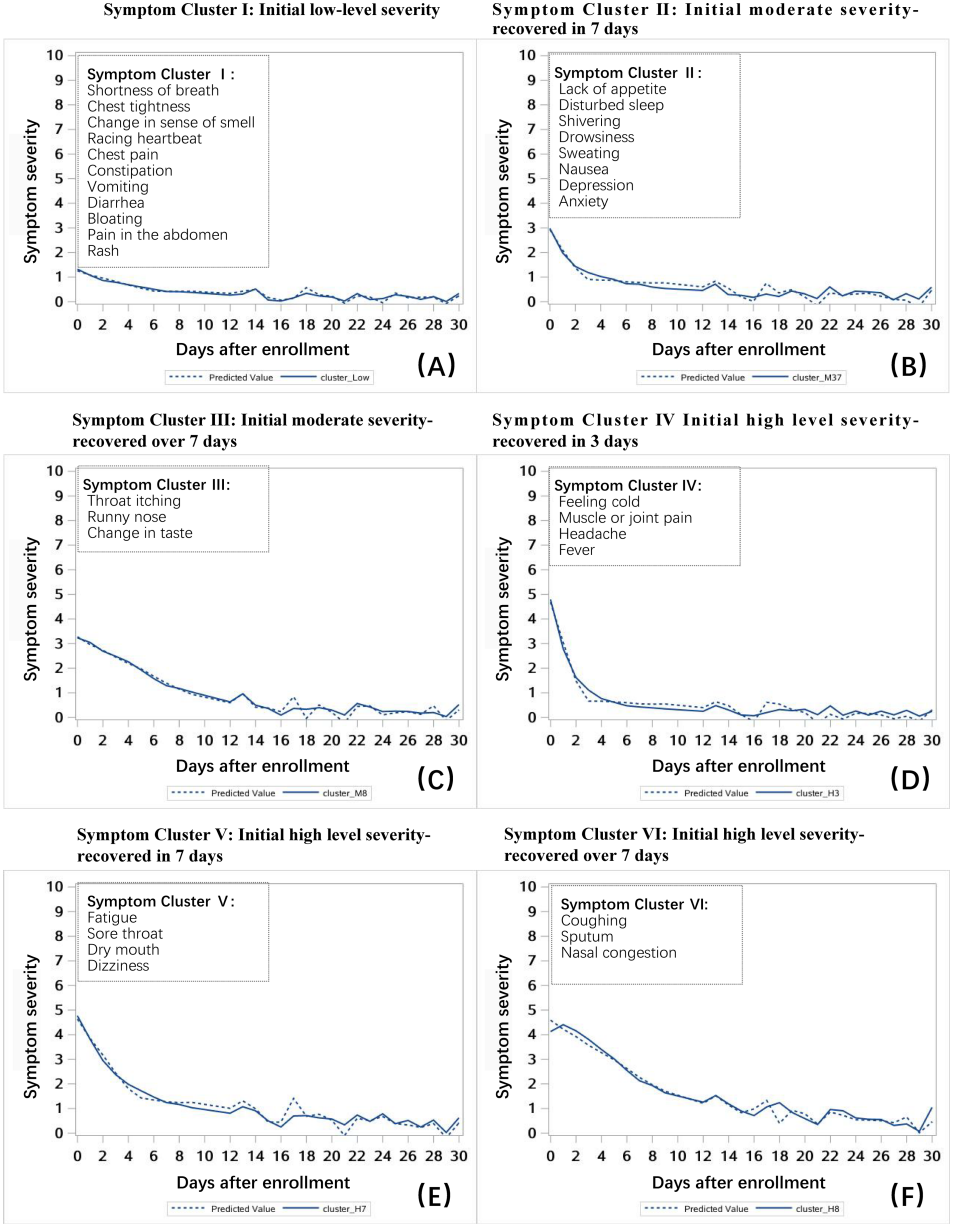
Symptom clusters identified using a mixed-effects model. **(A)** Symptom Cluster I: initial low severity (score <2). **(B)** Symptom Cluster II: initial moderate severity (score ≥2, <4) that improved within 7 days after enrollment. **(C)** Symptom Cluster III: initial moderate severity (score≥2, <4) that improved more than 7 days after enrollment. **(D)** Symptom Cluster IV: initial high severity (score ≥4) that improved within 3 days after enrollment. **(E)** Symptom Cluster V: initial high severity (score ≥4) that improved within 7 days after enrollment. **(F)** Symptom Cluster VI: initial high severity (score ≥4) that improved in more than 7 days after enrollment.

A total of 33 symptoms were clustered into six distinct symptom clusters, each representing a rehabilitation pattern (Figure. 2). In Symptom Cluster I, the severity of shortness of breath, chest tightness, change in sense of smell, racing heartbeat, chest pain, constipation, vomiting, diarrhea, bloating, pain in the abdomen, and rash was low (score <2) in the early disease phase and remained mild within 1-month post-enrollment. In Symptom Cluster II, moderately severe symptoms (score ≥2, <4) in the early disease phase, which consisted of lack of appetite, disturbed sleep, shivering, drowsiness, sweating, nausea, depression, and anxiety, resolved within 7 days after enrollment. In contrast, Symptom Cluster III included throat itching, runny nose, and change in taste, which resolved more than 7 days post-enrollment. In Symptom Cluster IV, symptoms with high severity (score ≥4) in the early disease phase, which included feeling cold, muscle or joint pain, headache, and fever, improved significantly within 3 days. In Symptom Cluster V, fatigue, sore throat, dry mouth, and dizziness improved significantly within 7 days. Finally, Symptom Cluster VI included coughing, sputum, and nasal congestion, which improved significantly more than 7 days after enrollment.

Table 3 presents patient and clinical factors predictive of the composite score for PRO symptom clusters. Compared with male patients, female patients were more likely to report higher severity levels for Symptom Cluster I and II (*p* < 0.05) and Symptom Cluster V and VI (*p* < 0.01). Patients who received more than three COVID-19 vaccine doses were more likely than those who received fewer than three doses to report lower severity levels for Symptom Cluster I (*p* < 0.01) and Symptom Cluster II and IV (*p* < 0.05). BMI ≥ 24 was a risk factor for higher severity levels of Symptom Cluster VI over time (*p* < 0.01). Longitudinally, patients with higher severity scores for Symptom Cluster II and Symptom Cluster V reported poorer quality of life than other clusters during the first month [estimate = −3.01 (SE = 0.81) and estimate = −2.41 (SE = 0.67), respectively; both *p* < 0.01].

**Table 3:** Influencing factors of symptom clusters.

Symptom cluster	Variable	Estimate	SD	DF	*t*	*p*
Symptom Cluster I: initial low severity (score <2)	Intercept	0.91	0.06	348	14.41	<0.01
	Days since recruitment	−0.02	0.001	342	−14.34	<0.01
	Symptom duration at enrollment	−0.02	0.01	3,450	−1.73	0.08
	Age	−0.001	0.001	3,450	−1.01	0.31
	Female sex	0.07	0.03	3,450	2.11	0.03
	BMI ≥ 24 kg/m^2^	−0.04	0.04	3,450	−1.04	0.30
	Comorbidities	0.25	0.05	3,450	5.27	<0.01
	3 or more vaccine doses received	−0.15	0.04	3,450	−4.16	<0.01
Symptom Cluster II: initial moderate severity (score ≥2, <4) that improved within 7 days after enrollment	Intercept	1.82	0.10	348	18.11	<0.01
	Days since recruitment	−0.03	0.002	342	−16.38	<0.01
	Symptom duration at enrollment	−0.09	0.02	3,450	−5.15	<0.01
	Age	−0.01	0.002	3,450	−3.06	<0.01
	Female sex	0.13	0.05	3,450	2.44	0.01
	BMI ≥ 24 kg/m^2^	−0.10	0.06	3,450	−1.82	0.07
	Comorbidities	0.31	0.08	3,450	4.08	<0.01
	3 or more vaccine doses received	−0.14	0.06	3,450	−2.40	0.02
Symptom Cluster III: initial moderate severity (score ≥2, <4) that improved more than 7 days after enrollment	Intercept	2.80	0.12	348	24.21	<0.01
	Days since recruitment	−0.06	0.002	342	−27.26	<0.01
	Symptom duration at enrollment	−0.08	0.02	3,450	−3.89	<0.01
	Age	−0.01	0.002	3,450	−5.18	<0.01
	Female sex	0.12	0.06	3,450	1.85	0.06
	BMI ≥ 24 kg/m^2^	0.02	0.06	3,450	0.34	0.73
	Comorbidities	0.09	0.09	3,450	1.06	0.29
	3 or more vaccine doses received	−0.04	0.07	3,450	−0.55	0.58
Symptom Cluster IV: initial high severity (score ≥4) that improved within 3 days after enrollment	Intercept	2.00	0.11	348	17.73	<0.01
	Days since recruitment	−0.04	0.002	342	−22.70	<0.01
	Symptom duration at enrollment	−0.12	0.02	3,450	−6.34	<0.01
	Age	−0.002	0.002	3,450	−0.88	0.38
	Female sex	0.07	0.06	3,450	1.18	0.24
	BMI ≥ 24 kg/m^2^	0.01	0.06	3,450	0.21	0.84
	Comorbidities	0.16	0.08	3,450	1.92	0.06
	3 or more vaccine doses received	−0.15	0.06	3,450	−2.33	0.02
Symptom Cluster V: initial high severity (score ≥4) that improved within 7 days after enrollment	Intercept	3.14	0.13	348	23.47	<0.01
	Days since recruitment	−0.06	0.003	342	−24.48	<0.01
	Symptom duration at enrollment	−0.17	0.02	3,450	−7.00	<0.01
	Age	−0.01	0.003	3,450	−3.10	<0.01
	Female sex	0.25	0.07	3,450	3.43	<0.01
	BMI ≥ 24 kg/m^2^	−0.01	0.07	3,450	−0.15	0.88
	Comorbidities	0.24	0.10	3,450	2.39	0.02
	3 or more vaccine doses received	−0.14	0.08	3,450	−1.89	0.06
Symptom Cluster VI: initial high severity (score ≥4) that improved in more than 7 days after enrollment	Intercept	4.31	0.14	348	30.59	<0.01
	Days since recruitment	−0.08	0.003	342	−30.47	<0.01
	Symptom duration at enrollment	−0.15	0.02	3,450	−6.08	<0.01
	Age	−0.02	0.003	3,450	−8.25	<0.01
	Female sex	0.25	0.08	3,450	3.33	<0.01
	BMI ≥ 24 kg/m^2^	0.22	0.08	3,450	2.76	<0.01
	Comorbidities	0.03	0.11	3,450	0.26	0.79
	3 or more vaccine doses received	0.004	0.08	3,450	0.04	0.97

Estimate, the solution vector for the fixed effect of the corresponding variable; DF, degrees of freedom.

## 4 Discussion

To the best of our knowledge, this is the first prospective longitudinal cohort study to report six distinct development and recovery profiles of multiple symptoms among non-hospitalized young adults during the Omicron variant-driven COVID-19 outbreak in China starting in late 2022. Symptom clusters were identified based on symptom severity rather than incidence, and we determined the symptom clusters that most affected quality of life, which was needed to alert clinicians to initiate prompt interventions. Early identification and precise management of these core symptoms in younger patients may help alleviate healthcare burden during future public health emergencies.

Although previous prospective studies have tracked COVID-19 symptoms before and after infection, they focused on the trajectory of single symptoms (14). Two other studies (15, 16) explored the evolution of symptom clusters of wild-type, alpha-variant, and delta-variant based on the incidence or frequency of symptoms. In contrast, we focused on the severity of symptoms and identified Symptom Cluster II and Symptom Cluster V as the core symptoms that most affect the quality of life of patients. In addition, the participants in our study had not been infected with SARS-CoV-2 previously. They were mainly younger than 60 years, and most of them had no comorbidities. Thus, our findings regarding the evolution of their symptoms may clearly reflect the characteristics of primary infection with the Omicron subvariants.

In the present study, participants presented with a substantial symptom burden at COVID-19 onset. Coughing, sore throat, and sputum were the most common symptoms at enrollment, suggesting a direct impact of the Omicron subvariants on the respiratory system. Previous studies demonstrated that the most common symptoms during the Omicron outbreak were sore throat and coughing (17, 18). However, we observed a higher prevalence of these two symptoms compared with previous reports. Fernandez-de-Las-Peñas et al. (19) performed a meta-analysis of symptoms in the general population 2 years after SARS-CoV-2 infection, which showed that fatigue was the most prevalent post-COVID symptom. Luo et al. (20) conducted a meta-analysis of persistent symptom prevalence over a 4-week to 24-month follow-up period, which indicated that fatigue, dyspnea, post-traumatic stress disorder, anxiety, and depression were the five most prevalent symptoms. These studies involved long-term follow-up of symptoms following wild-type SARS-CoV-2 infection, but these investigations only assessed symptom incidence and failed to indicate which symptoms warranted clinical attention, as moderate-to-severe symptoms are more meaningful for clinical intervention. Our research shows that fatigue was the most prevalent symptom and ranked highest in terms of moderate to severe incidence at enrollment to 9 months of follow-up. Depression and anxiety related to emotion were among the top five most frequently occurring symptoms during the 3–9 months follow-up period, and were also among the top five in terms of the incidence of moderate-to-severe symptoms.

Prior research findings demonstrated that recovery was slower in female compared to male participants and those with a BMI ≥ 30 kg/m^2^ compared to BMI < 25 kg/m^2^ (21). Additionally, women more often reported the presence of ≥3 persistent post-acute COVID-19 symptom (22). In our study, we also observed the influence of both gender and BMI on long-term COVID symptoms. We found that female patients and those with a BMI ≥ 24 were more likely to report a higher severity level of long-term symptoms. This further confirms the crucial role of gender and BMI in the context of chronic health issues. It suggests that in subsequent medical interventions and health management, more attention should be placed on these groups.

Multiple symptom clusters were more severe in younger participants than in older adults, which is inconsistent with previous findings (23, 24). This discrepancy may be attributed to a more robust inflammatory response in younger individuals post-infection. It may also be related to the fact that most of the patients in this study were younger than 60 years, suggesting limitations in terms of the representativeness of the study. We also found that receiving more than three COVID-19 vaccine doses protected patients by reducing the severity of their febrile symptoms and autonomic dysfunction, which is consistent with prior research (25). Antonelli et al. (25) showed that during the Omicron outbreak, self-reported symptoms (e.g., fever, headache, feeling cold, muscle pain, shivering, and nausea) were less frequent among individuals who received three vaccine doses compared with those who received two doses.

Our study had several strengths. First, we used a validated 0–10 numeric scale to quantify symptom severity. Second, our prospective longitudinal design captured symptoms in real time, minimizing recall bias and showing the trajectory of symptom evolution more clearly than cross-sectional surveys. Third, an electronic patient-reported outcome (ePRO) portal enhanced data accuracy and reliability. Finally, whereas researchers in previous studies mainly observed the relationships of demographic characteristics with prognosis for COVID-19 and mainly focused on hospital admission rates and mortality, we focused on the relationships among symptom clusters, duration, and severity and patient health over the full course of SARS-CoV-2 infection. Several limitations of this study should be acknowledged. First, we did not recruit uninfected subjects for comparison with infected patients. Second, classification as Omicron variant infection was based on prevailing epidemic timeline rather than individual variant confirmation. Third, lack of clinician-reported data may have led to loss of information about patients’ symptoms. Fourth, using ePRO could have biased our results toward young persons and might have limited participation by less technologically literate persons and persons without internet at home.

## 5 Conclusion

Our results reflect the types, duration, and severity of COVID-19 symptoms, demonstrating differences in the symptom characteristics of patients with different baseline characteristics, characterizing the differences in health states over the full disease course in COVID-19 patients with different symptom characteristics, and identifying the symptom clusters that most affected quality of life. Taken together, our findings represent a valuable reference for the management of COVID-19 symptoms.

## Data Availability

The raw data supporting the conclusions of this article will be made available by the authors, without undue reservation.

## References

[1] GBD 2021 Demographics Collaborators. Global age-sex-specific mortality, life expectancy, and population estimates in 204 countries and territories and 811 subnational locations, 1950–2021, and the impact of the COVID-19 pandemic: a comprehensive demographic analysis for the global burden of disease study 2021. Lancet. (2024) 403:1989–2056. doi: 10.1016/S0140-6736(24)00476-8 38484753 PMC11126395

[2] Ely EW Brown LM Fineberg HV. Long covid defined. N Engl J Med. (2024) 391:1746–53. doi: 10.1056/NEJMsb2408466, 39083764 PMC11687645

[3] Van Wambeke E Bezler C Kasprowicz AM Charles AL Andres E Geny B. Two-years follow-up of symptoms and return to work in complex post-COVID-19 patients. J Clin Med. (2023) 12:741. doi: 10.3390/jcm12030741, 36769389 PMC9917586

[4] Qin S Zhang Y Li Y Huang L Yang T Si J . Long COVID facts and findings: a large-scale online survey in 74,075 Chinese participants. Lancet Reg Health West Pac. (2024) 52:101218. doi: 10.1016/j.lanwpc.2024.101218, 39881668 PMC11776084

[5] Miaskowski C Dodd M Lee K. Symptom clusters: the new frontier in symptom management research. J Natl Cancer Inst Monogr. (2004) 32:17–21. doi: 10.1093/jncimonographs/lgh023, 15263036

[6] El-Shabasy RM Nayel MA Taher MM Abdelmonem R Shoueir KR Kenawy ER. Three waves changes, new variant strains, and vaccination effect against COVID-19 pandemic. Int J Biol Macromol. (2022) 204:161–8. doi: 10.1016/j.ijbiomac.2022.01.118, 35074332 PMC8782737

[7] Wu ZY McGoogan JM. Characteristics of and important lessons from the coronavirus disease 2019 (COVID-19) outbreak in China: summary of a report of 72 314 cases from the Chinese Center for Disease Control and Prevention. JAMA. (2020) 323:1239–42. doi: 10.1001/jama.2020.2648 32091533

[8] Augustin M Schommers P Stecher M Dewald F Gieselmann L Gruell H . Post-COVID syndrome in non-hospitalised patients with COVID-19: a longitudinal prospective cohort study. Lancet Reg Health Eur. (2021) 6:100122. doi: 10.1016/j.lanepe.2021.100122, 34027514 PMC8129613

[9] Chen C Haupert SR Zimmermann L Shi X Fritsche LG Mukherjee B. Global prevalence of post-coronavirus disease 2019 (COVID-19) condition or long COVID: a meta-analysis and systematic review. J Infect Dis. (2022) 226:1593–607. doi: 10.1093/infdis/jiac136, 35429399 PMC9047189

[10] Fernández-de-las-Peñas C Rodríguez-Jiménez J Cancela-Cilleruelo I Guerrero-Peral A Martín-Guerrero JD García-Azorín D . Post-COVID-19 symptoms 2 years after SARS-CoV-2 infection among hospitalized vs non-hospitalized patients. JAMA Netw Open. (2022) 5:e2242106. doi: 10.1001/jamanetworkopen.2022.42106, 36378309 PMC9667330

[11] U.S. Department of Health and Human Services FDA Center for Drug Evaluation and Research, U.S. Department of Health and Human Services FDA Center for Biologics Evaluation and Research, U.S. Department of Health and Human Services FDA Center for Devices and Radiological Health. Guidance for industry: patient-reported outcome measures: use in medical product development to support labeling claims: draft guidance. Health Qual Life Outcomes. (2006) 4:79. doi: 10.1186/1477-7525-4-79, 17034633 PMC1629006

[12] Zhang J Guo Q Chen J Liu Y Kang D Xiang R . An electronic patient-reported outcome symptom monitor: the Chinese experience with rapid development of a ready-to-go symptom monitor. BMC Public Health. (2024) 24:2989. doi: 10.1186/s12889-024-20518-5, 39472836 PMC11520658

[13] von Elm E Altman DG Egger M Pocock SJ Gøtzsche PC Vandenbroucke JP. The strengthening the reporting of observational studies in epidemiology (STROBE) statement: guidelines for reporting observational studies. J Clin Epidemiol. (2008) 61:344–9. doi: 10.1016/j.jclinepi.2007.11.008, 18313558

[14] Ballering AV van Zon SKR Olde Hartman TC Rosmalen JGM Lifelines Corona Research Initiative. Persistence of somatic symptoms after COVID-19 in the Netherlands: an observational cohort study. Lancet. (2022) 400:452–61. doi: 10.1016/S0140-6736(22)01214-4, 35934007 PMC9352274

[15] Canas LS Molteni E Deng J Sudre CH Murray B Kerfoot E . Profiling post-COVID-19 condition across different variants of SARS-CoV-2: a prospective longitudinal study in unvaccinated wild-type, unvaccinated alpha-variant, and vaccinated delta-variant populations. Lancet Digit Health. (2023) 5:e421–34. doi: 10.1016/S2589-7500(23)00056-0, 37202336 PMC10187990

[16] Wiegele PN Kabar I Kerschke L Froemmel C Hüsing-Kabar A Schmidt H . Symptom diary-based analysis of disease course among patients with mild coronavirus disease, Germany, 2020. Emerg Infect Dis. (2021) 27:1353–61. doi: 10.3201/eid2705.204507, 33900166 PMC8084503

[17] Menni C Valdes AM Polidori L Antonelli M Penamakuri S Nogal A . Symptom prevalence, duration, and risk of hospital admission in individuals infected with SARS-CoV-2 during periods of omicron and delta variant dominance: a prospective observational study from the ZOE COVID study. Lancet. (2022) 399:1618–24. doi: 10.1016/S0140-6736(22)00327-0, 35397851 PMC8989396

[18] Yang H Wang Z Zhang Y Xu M Wang Y Zhang Y . Clinical characteristics and factors for serious outcomes among outpatients infected with the omicron subvariant BF.7. J Med Virol. (2023) 95:e28977. doi: 10.1002/jmv.28977, 37635385

[19] Fernandez-de-Las-Peñas C Notarte KI Macasaet R Velasco JV Catahay JA Ver AT . Persistence of post-COVID symptoms in the general population two years after SARS-CoV-2 infection: a systematic review and meta-analysis. J Infect. (2024) 88:77–88. doi: 10.1016/j.jinf.2023.12.004 38101521

[20] Luo D Mei B Wang P Li X Chen X Wei G . Prevalence and risk factors for persistent symptoms after COVID-19: a systematic review and meta-analysis. Clin Microbiol Infect. (2024) 30:328–35. doi: 10.1016/j.cmi.2023.10.016, 37866679

[21] Wynberg E van Willigen HDG Dijkstra M Boyd A Kootstra NA van den Aardweg JG . Evolution of coronavirus disease 2019 (COVID-19) symptoms during the first 12 months after illness onset. Clin Infect Dis. (2022) 75:e482–90. doi: 10.1093/cid/ciab759, 34473245 PMC8522402

[22] Ghosn J Bachelet D Livrozet M Cervantes-Gonzalez M Poissy J Goehringer F . Prevalence of post-acute coronavirus disease 2019 symptoms twelve months after hospitalization in participants retained in follow-up: analyses stratified by gender from a large prospective cohort. Clin Microbiol Infect. (2023) 29:254.e7–254.e13. doi: 10.1016/j.cmi.2022.08.028 PMC9523945 36191847

[23] Chenchula S Vidyasagar K Pathan S Sharma S Chavan MR Bhagavathula AS . Global prevalence and effect of comorbidities and smoking status on severity and mortality of COVID-19 in association with age and gender: a systematic review, meta-analysis and meta-regression. Sci Rep. (2023) 13:6415. doi: 10.1038/s41598-023-33314-9, 37076543 PMC10115382

[24] Larsson SB von Feilitzen GS Andersson ME Sikora P Lindh M Nordén R . Self-reported symptom severity, general health, and impairment in post-acute phases of COVID-19: retrospective cohort study of Swedish public employees. Sci Rep. (2022) 12:19818. doi: 10.1038/s41598-022-24307-1, 36396860 PMC9672032

[25] Antonelli M Penfold RS Canas LDS Sudre C Rjoob K Murray B . SARS-CoV-2 infection following booster vaccination: illness and symptom profile in a prospective, observational community-based case-control study. J Infect. (2023) 87:506–15. doi: 10.1016/j.jinf.2023.08.009, 37777159 PMC7618294

